# Prognostic Characteristics of Metabolic Dysfunction-Associated Steatotic Liver in Patients with Obesity Who Undergo One Anastomosis Gastric Bypass Surgery: A Secondary Analysis of Randomized Controlled Trial Data

**DOI:** 10.3390/nu16183210

**Published:** 2024-09-23

**Authors:** Silke Crommen, Karl Peter Rheinwalt, Andreas Plamper, Daniela Rösler, Leonie Weinhold, Christine Metzner, Sarah Egert

**Affiliations:** 1 Institute of Nutritional and Food Sciences, Nutritional Physiology, University of Bonn, 53113 Bonn, Germany; s.crommen@uni-bonn.de; 2 Department of Bariatric, Metabolic and Plastic Surgery, Cellitinnen-Krankenhaus St. Franziskus Cologne, 50825 Cologne, Germany; karlpeter.rheinwalt@cellitinnen.de (K.P.R.); andreas.plamper@cellitinnen.de (A.P.); 3 Bonn Education Association for Dietetics r.A., 50935 Cologne, Germany; office@bfdev.de (D.R.); christine.metzner@rwth-aachen.de (C.M.); 4 Institute of Medical Biometry, Informatics and Epidemiology, University Hospital Bonn, 53127 Bonn, Germany; weinhold@imbie.uni-bonn.de; 5 Medical Clinic III—Department of Gastroenterology, Metabolic Disorders and Internal Intensive Care, University Hospital RWTH Aachen, 52074 Aachen, Germany

**Keywords:** bariatric surgery, mini gastric bypass, one anastomosis gastric bypass, metabolic dysfunction-associated steatotic liver disease, nonalcoholic fatty liver disease, insulin resistance

## Abstract

Background/Objectives: Metabolic dysfunction-associated steatotic liver disease (MASLD) is closely associated with obesity and insulin resistance (IR). Identifying characteristics that predict a higher risk of fibrosis using noninvasive methods is particularly important. Methods: We performed a secondary analysis of data from an RCT of 48 patients after one anastomosis gastric bypass (OAGB) surgery, supplemented with specifically formulated probiotics and micronutrients or control treatment for 12 weeks. Patients were categorized using alanine aminotransferase (ALAT; >35 U/L for women, >50 U/L for men), higher NAFLD fibrosis score (NFS) > −1.455), and IR (HOMA-IR > 2.0). This trial was registered at Clinicaltrials.gov (ID: NCT03585413). Results: Abnormal ALAT was associated with high triglycerides, blood pressure (BP), glucose, and fatty liver index (FLI). NFS > −1.455 was linked to higher age, body mass, waist circumference, and FLI, and lower albumin and platelet count. HOMA-IR > 2.0 was associated with higher BP and triglycerides, lower HDL-cholesterol, higher serum transaminases, and higher probabilities of steatosis and fibrosis. Twelve weeks postoperatively, patients with NFS > −1.455 showed greater reductions in body mass, systolic BP, serum insulin, and HbA1c, whereas those with NFS ≤ −1.455 showed improvements in FLI and lipid metabolism but had high glucose concentrations. Patients with HOMA-IR ≤ 2.0 also had high glucose concentrations. Conclusions: The evaluation of common biomarker scores for fibrosis and IR may help clinicians to recognize severe NAFLD and improve the outcomes of OAGB surgery.

## 1. Introduction

Metabolic dysfunction-associated steatotic liver disease (MASLD), previously termed nonalcoholic fatty liver disease (NAFLD), became the most common cause of chronic liver disease. It is frequently associated with visceral obesity, hypertension, insulin resistance (IR), and dyslipoproteinemia, and is often regarded as the hepatic manifestation of the metabolic syndrome (MetS) [1,2]. Indeed, type 2 diabetes mellitus (T2DM) is a major risk factor for the progression of metabolic dysfunction-associated steatotic liver (MASL) toward metabolic dysfunction-associated steatohepatitis (MASH), cirrhosis, and hepatocellular carcinoma [3]. Liver biopsy is the gold standard method of diagnosing MASLD and is required for the staging of the disease. However, it is an invasive technique, and may be accompanied by complications, such as hemorrhage and liver hematoma, puncture of the gall bladder or nearby organs, and hemo- or pneumothorax [4,5]. Given the substantial risks of morbidity and mortality, timely recognition of MASH is imperative [6]. Therefore, noninvasive serum/blood tests, including the measurement of routine biochemical markers, were established for the assessment of hepatic steatosis and fibrosis. For example, high circulating aminotransferase activities, along with the presence of T2DM, were shown to be independent diagnostic biomarkers of fibrosis in patients with MASLD [7]. Moreover, a high alanine aminotransferase (ALAT) activity in particular is associated with higher risks of T2DM and MetS [8]. The aminotransferases ALAT and aspartate aminotransferase (ASAT) were used in several scoring systems for liver diseases, such as the NAFLD fibrosis score (NFS), which was developed to estimate the risk of advanced fibrosis in patients with MASLD [9]. Each of the variables included in the NFS (age, body mass index (BMI), ALAT, ASAT, platelet count, albumin concentration, and diabetes) is associated with a high risk of systemic metabolic disturbance.

In general, bariatric surgery yielded impressive results with respect to the resolution of features of MASLD [10,11,12], but there remain concerns regarding this technique, owing to transient post-surgical increases in serum liver enzyme activities and the progression of fibrosis in a subset of patients, especially after gastric bypass procedures [13]. Moreover, bariatric surgery can improve glycemic control in patients with T2DM within days of surgery. However, there are only superficial studies of patients with both MASLD and T2DM to date. Thus, there is a need to elucidate the specific metabolic effects of bariatric surgery on individuals with obesity and T2DM, in addition to MASLD, because these comorbidities are associated with a high risk of MASLD progression.

In our recent study, we demonstrated that 12 weeks of dietary supplementation with a specifically tailored probiotic and micronutrient preparation yielded significant improvements in patients with obesity after one anastomosis gastric bypass (OAGB), previously termed mini gastric bypass, surgery. Compared with the control treatment of placebo powder and basic care micronutrients, the supplemented group had lower serum triglyceride concentrations, superior visceral tissue function indicated by an improvement in visceral adiposity index (VAI), lower serum ASAT, and lower NFS. These findings demonstrate that targeted probiotic and micronutrient supplementation after OAGB surgery mitigates liver injury [14].

In the present study, we performed a secondary analysis of data collected during our randomized controlled clinical trial to identify predictors of abnormal ALAT, as the first index of the transition from MASL to MASH and higher risks of fibrosis and IR, in patients with obesity after OAGB surgery. In addition, we aimed to assess the implications of these higher probabilities of fibrosis and IR on the outcomes of bariatric surgery. The in-depth analysis of these patients provides insight into the characteristics of patients with MASLD who are at a high risk of MASH or advanced fibrosis and the metabolic effects of bariatric surgery. We hypothesized that the NFS of patients at baseline would influence their cardiometabolic parameters post OAGB surgery.

## 2. Materials and Methods

We performed a secondary analysis of data collected during an RCT [14].

### 2.1. Participants and Study Design

Patients who were scheduled to undergo OAGB surgery were recruited at the Department of Bariatric, Metabolic, and Plastic Surgery, Cellitinnen-Krankenhaus St. Franziskus, Cologne, Germany. The study design, and of patient recruitment, enrolment, and randomization, were described previously [14]. Briefly, interested patients (*n* = 78) aged 20–65 years underwent screening that included physical examinations, laboratory testing, the taking of a medical history, and a dietary habit assessment. Patients were included if they had a BMI of 35.0–49.9 kg/m^2^, were candidates for primary laparoscopic OAGB, and had a fatty liver index (FLI) > 60, indicative of hepatic steatosis [15].

All the study procedures were approved by the Freiburg International Ethics Commission (approval number 02018/1161; date of approval 12 March 2018). Written informed consent was obtained from all the patients prior to their inclusion. The trial was prospectively registered at Clinicaltrials.gov (ID: NCT03585413). The data collected for 48 patients, who were retrospectively divided into low- and higher-risk groups with respect to fibrosis and IR, were analyzed.

A randomized, double-blind, controlled trial was performed as follows. Patients undergoing OAGB surgery were administered either a specifically tailored probiotic and micronutrient combination (Pro + SM) or a placebo and basic micronutrient treatment (Con + BM) for 12 weeks. A detailed description of the treatments is presented in Crommen et al. [14]. The probiotic or placebo powder was taken twice daily and the micronutrient capsule was taken three times daily with meals. The probiotic and placebo had similar appearance, texture, taste, and smell. Patients were assigned to the treatment groups by block randomization, and the assignment was not disclosed to the surgical, medical, or nutritional staff, nor to the investigators nor the patients themselves.

### 2.2. Surgical Technique

All the laparoscopic OAGB procedures were performed by a surgical team consisting of three surgeons. The technique was described previously [16]. In brief, OAGB surgery entailed the formation of a pouch along the minor gastric curvature, while maintaining a standardized biliopancreatic limb of 200 cm in length for all the patients, based on the recommendations of Lee et al. [17]. As part of standard perioperative care, the patients were prescribed preoperative antibiotics (metronidazole 500 mg and cephalosporine on the basis of body mass), 4 weeks of low-molecular weight heparin (dalteparin, 5000 IU/d), and 3 months of a proton pump inhibitor (pantoprazole, 40 mg/d).

### 2.3. Blood Sample Processing and Analysis

The pre-analytical procedures used for blood samples were described previously [14]. NFS was calculated in wk 0 and after the 12 wk intervention period, using the following formula: NFS = −1.675 + 0.037 × age (years) + 0.094 × BMI (kg/m^2^) + 1.13 × impaired fasting glucose/diabetes (yes = 1, no = 0) + 0.99 × ASAT/ALAT ratio − 0.013 × platelet count (×10^9^/L) − 0.66 × albumin (g/dL). Fibrosis was staged according to the cut-off points proposed by Angulo et al. [9] as follows: no fibrosis (NFS < −1.455), intermediate fibrosis risk (NFS > −1.455), and the presence of fibrosis (NFS > 0.676). HOMA-IR was calculated and used as a surrogate for IR, with a HOMA-IR of >2.0 being considered indicative of IR [18].

### 2.4. Statistical Analyses

Data were analyzed using SPSS version 27 (IBM Corporation, Armonk, NY, USA). Owing to the low number of patients with NFS > 0.676, the patients were divided into those with a baseline NFS < −1.455 or >−1.455, as well as into those with an ALAT activity below or above the reference value (35 U/L for women, 50 U/L for men), to permit a comparison of patients who were more or less likely to have a high risk of MASLD. The characteristics of the patients are expressed as frequencies and percentages for categorical data and as the arithmetic mean ± standard deviation for continuous data.

To compare continuous variables between the NFS groups (≤−1.455 vs. >−1.455), HOMA-IR groups (<2.0 vs. ≥2.0), and ALAT groups (normal vs. abnormal), the independent samples *t*-test was used. To investigate the longitudinal changes of biomarkers, dependent samples *t*-tests were used. The comparison of changes in variables between HOMA-IR and NFS groups was carried out using independent samples *t*-tests. All the tests were two-tailed, and *p* ≤ 0.05 was considered statistically significant. Unless otherwise stated, data are presented as the arithmetic mean ± standard deviation (SD). All the presented analyses were exploratory, and *p*-values were not adjusted for multiple testing.

## 3. Results

The recruitment of the patients and the baseline results were previously published [14]. A total of 60 patients (10 men and 50 women) were randomly enrolled. Twelve patients were subsequently excluded for various reasons: a failure in venous blood sample collection (*n* = 1), withdrawal from the study (*n* = 6), antibiotic treatment (*n* = 3), and non-compliance with treatment instructions (*n* = 2).

The baseline characteristics of the entire study group are shown in Table 1. At wk 0, the mean age of the patients as a whole was 40.1 ± 9.6 years, they had a mean preoperative body mass and BMI of 123.9 ± 16.1 kg and 43.2 ± 3.3 kg/m^2^, respectively (Table 1). Of these patients, 27% were known to have T2DM prior to surgery. According to the International Diabetes Federation (IDF) criteria, 85% of the patients were classified as having metabolic syndrome. At week 0, all the patients had an FLI above the cut-off value of 60, indicating the presence of steatosis, and 60% the patients (29 of 48) had an NFS of >−1.455, indicative of an intermediate probability of advanced fibrosis. NFS values above 0.676, indicating advanced fibrosis, were found in two patients (Table 1).

As expected, the mean age, body mass, BMI, waist circumference, and FLI of patients with NFS > −1.455 were higher than those of the patients with NFS ≤ −1.455 (all *p* < 0.05, Table 1). Furthermore, NFS > −1.455 was associated with lower serum albumin and platelet count. There were no significant differences in markers of inflammation or in the blood pressure (BP) of the patients at a low or higher risk of fibrosis (all *p* > 0.05, Table 1).

In wk 0, three patients (11%) with HOMA-IR < 2.0 were known to have T2DM, whereas the group of patients with HOMA-IR ≥ 2.0, 10 (50%) were known to have T2DM. Patients with a HOMA-IR ≥ 2.0 were older, heavier, and had high systolic and diastolic BP (all *p* < 0.05, Table 2). Furthermore, high HOMA-IR was associated with larger waist circumference, higher triglyceride concentration and VAI, and lower HDL-cholesterol concentration (all *p* < 0.05, Table 2). By contrast, there was no difference in the fat mass of the patients with HOMA-IR values lower or higher than the cut-off of 2.0. As expected, the circulating glucose and insulin concentrations and the HbA1c of patients with HOMA-IR ≥ 2.0 were higher than those with HOMA-IR < 2.0. HOMA-IR ≥ 2.0 was associated with higher transaminase activities, lower ASAT/ALAT ratio, and a higher risk of fibrosis (all *p* < 0.05, Table 2).

Patients with abnormal ALAT activities had higher waist circumferences and triglyceride concentrations than those with normal activities (all *p* < 0.05, Table 3). Furthermore, abnormal ALAT was associated with higher systolic and diastolic BP, glucose concentration, transaminase activities, GGT activity, FLI, and ferritin concentration; and lower ASAT/ALAT ratio and serum albumin concentration (all *p* < 0.05, Table 3).

Twelve weeks after OAGB surgery, patients with NFS > −1.455 showed larger decreases in body mass, systolic BP, serum insulin concentration, and HbA1c than those with NFS ≤ −1.455 (all *p* < 0.05, Table 4). By contrast, patients with NFS ≤ −1.455 showed larger decreases in FLI and LDL and total cholesterol concentrations than those with NFS > −1.455 (all *p* < 0.05; Table 4). Treatment-specific evaluation of the data did not yield any additional findings (Table S1).

During the study, the patients with HOMA-IR < 2.0 showed increases in fasting glucose concentration and HOMA-IR, whereas those with HOMA-IR ≥ 2.0 showed an improvement in HbA1c. The fasting glucose and insulin concentrations, HOMA-IR, and HbA1c of patients with HOMA-IR ≥ 2.0 improved vs. those of patients with HOMA-IR < 2.0 (all *p* < 0.05, Table 5). Furthermore, patients with HOMA-IR ≥ 2.0 showed larger reductions in serum ferritin concentration than those with HOMA-IR < 2.0 (Table 5). Patients with HOMA-IR < 2.0 also showed a larger reduction in FLI than those with HOMA-IR ≥ 2.0 (*p* < 0.05, Table 5). Treatment-specific evaluation of the data did not yield any additional findings (Table S2).

## 4. Discussion

We described a secondary analysis of data collected during a double-blind, randomized controlled clinical trial of 48 patients with morbid obesity who underwent OAGB surgery. The major finding was that an abnormal serum ALAT activity is associated with high triglyceride concentration, systolic and diastolic BP, fasting glucose concentration, and FLI. A higher risk of fibrosis was linked to older age, higher body mass, larger waist circumference, higher FLI, and lower albumin concentration and platelet count. In addition, HOMA-IR > 2.0, indicative of IR, was associated with high systolic and diastolic BP, high triglyceride concentration, low HDL-cholesterol concentration, high serum transaminase activities, and higher risks of fatty liver and fibrosis. Twelve weeks after OAGB surgery, patients with a high risk of fibrosis showed larger reductions in body mass, systolic BP, serum insulin concentration, and HbA1c than those with a lower risk.

The natural course of MASLD varies, with most patients having simple steatosis and not progressing to severe fibrosis and liver disease. However, those with progressive MASLD, who develop MASH and/or advanced fibrosis, along with those who also have T2DM, are at the highest risks of developing cardiovascular disease (CVD) [19]. Therefore, ectopic liver fat appears to be a key driver of adverse cardiometabolic outcomes. Given the increasing recognition of the associations of MASLD with cardiovascular and all-cause mortality, it is particularly important to identify fibrosis at an early stage and to monitor and manage this fibrosis to mitigate the risks of adverse outcomes. The recent change in terminology from NAFLD to MASLD also highlights the link between hepatic steatosis and metabolic dysfunction. This reclassification should enable better identification of subjects at higher risk of overall and cardiovascular mortality, leading to improved patient outcomes [20,21]. The circulating activities of liver-derived transaminases are widely recognized to be biochemical markers of MASLD. Indeed, previous reports and guidelines recommended that the measurement of liver transaminase activities should be used as the initial screening method for MASLD [22]. The primary focus of the present study was to evaluate the implications of a high ALAT activity because this enzyme is predominantly located in the cytoplasm of hepatocytes and is considered to be relatively specific for hepatic insults and injury [23]. High ALAT activity has good specificity for the diagnosis of MASLD, and several previous studies demonstrated that a high ALAT activity is associated with a higher risk of MASH [9,24]. Although there is no well-defined upper limit for the normal range of ALAT activity, 40 U/L is often used as the cut-off value for both men and women [25]. In addition, high serum ALAT activity was linked to higher risks of T2DM, cardiovascular disease, and MetS. Interestingly, high enzyme activity, even when within the normal range, is now thought to be associated with a higher risk of developing T2DM and MetS [26]. The present findings confirm a link between serum ALAT activity and T2DM status. At baseline, individuals with high ALAT activity had higher glucose concentrations and tended to have higher insulin concentrations and HOMA-IR scores than those with lower activity. Furthermore, consistent with the fact that IR and T2DM are the predominant risk factors for MASH and its progression to advanced fibrosis, and these are particularly prevalent in patients with obesity, we found that HOMA-IR influences serum liver transaminase activities and NFS. This bidirectional connection between MASLD and diabetes is well established [27]. IR contributes to MASLD directly by increasing de novo lipogenesis and indirectly by increasing free fatty acid flux to the liver via a decrease in the inhibition of lipolysis. Thus, a high HOMA-IR, an index that is easy to calculate and widely used in clinical practice, should raise suspicion regarding the presence of MASH, and highlight the need for further investigation and treatment.

Waist circumference reflects abdominal fat mass. MASLD is the hepatic component of MetS and is associated with abdominal visceral adiposity. It probably develops as a result of the visceral fat lipolysis, which is more marked than subcutaneous fat. Accordingly, we found that a large waist circumference was associated with a higher probability of advanced fibrosis. Interestingly, the fat mass of patients at low or higher risk of IR and MASLD fibrosis did not differ. The possible explanations for this include the relationship being lost with an increase in fat mass and the fact that subcutaneous fat predominated in the present patients. More important than the quantity of adipose tissue may be the quality and distribution, which we did not assess in detail. Therefore, future studies should also include measurements of organ fat content. However, in summary, in the present cohort of patients with high fat mass, fat mass alone was not an effective predictor of the risk of fibrosis.

In the present cohort, there were no differences in the serum triglyceride concentrations of patients with a low or higher probability of fibrosis. Moreover, waist circumference did not correlate with triglyceride concentration, suggesting that patients did not have a hypertriglyceridemic waist phenotype, which is surprising, given that the central components of MetS were present in these patients.

Moreover, we found that older age was associated with a higher probability of fibrosis. This finding was expected, because age is also included in the calculation of the NFS and the components of MetS become more prevalent and more marked with age [28,29]. In addition, women have a higher risk of advanced fibrosis than men, especially after the age of 50 years [30,31].

OAGB surgery ameliorated components of MetS and the cardiometabolic risk profile during the first 12 wk postoperatively. There is conflicting evidence about the effect of pre-existing MASLD on postoperative weight loss. One previous study demonstrated no significant correlation between the improvement in NAFLD score and the extent of weight loss [32], whereas Abu-Rumaileh et al. [33] found that patients with pre-existing MASLD had significantly lower total and excess weight loss than those without. This is in contrast to the results of the present study, in which patients with higher probability of fibrosis achieved greater weight loss than those with a lower probability of fibrosis. Moreover, patients with a higher probability of fibrosis showed larger decreases in systolic BP and HbA1c. By contrast, patients with a lower probability of fibrosis showed a larger reduction in FLI and an improvement in lipid metabolism.

Twelve weeks after surgery, patients with a high probability of fibrosis still had high ALAT activities, whereas those with a low probability showed the normalization of activity. Intraoperative biopsy remains the gold standard method of diagnosing and staging MASLD, but it is not always feasible to repeat this postoperatively. Therefore, serum aminotransferase activities should be measured regularly, and patients with known preoperative MASLD who do not show complete normalization of their liver enzyme activities or show repeated evidence of chronic hepatitis postoperatively should continue to be monitored for the progression of MASLD.

Bariatric surgery was shown to be more effective than standard medical care for the treatment of T2DM, as shown in randomized clinical trials lasting 1 to 5 years [34,35,36,37]. It greatly improves glycemic control, often within days of surgery, independently of weight loss. However, unexpectedly, patients with a lower probability of fibrosis experienced increases in fasting glucose concentrations during the 12 wk following OAGB surgery. Similar results were obtained when the HOMA indices of the patients were evaluated.

The main strength of the present study was that we studied a well-characterized sample of patients that was clinically relevant: patients with morbid obesity who underwent OAGB surgery. One limitation of the present work was that it was a secondary data analysis, and therefore exploratory in nature. The study was originally designed to investigate the effects of a specifically tailored probiotic and micronutrient mixture on MASLD-related indices; therefore, the data presented are the findings of complementary analyses. Another limitation was the relatively small number of patients studied.

## 5. Conclusions

Overall, the results of the present study highlight the importance of using simple noninvasive methods for the prognosis of liver disease, such as laboratory tests and indices (ALAT, NFS, and HOMA-IR), along with easily obtained anthropometric data (waist circumference) and the patient’s age. A combination of these indices may help to predict the risk of MASH in patients with extreme obesity, and close monitoring postoperatively may improve the outcomes of OAGB surgery.

## Figures and Tables

**Table 1:** Baseline anthropometric and clinical characteristics of the patients grouped using a NAFLD fibrosis score (NFS) cut-off value of −1.455 ^1^.

	All (*n* = 48)	NFS ≤ −1.455 (*n* = 17)	NFS > −1.455 (*n* = 31) *	*p*-Value ^2^
Age (years)	40.1 ± 9.6	35.4 ± 10.1	42.7 ± 8.3	0.010
Height (m)	1.69 ± 0.09	1.67 ± 0.07	1.70 ± 0.09	0.345
Body mass (kg)	123.9 ± 16.1	116.5 ± 11.2	128.0 ± 17.1	0.017
BMI (kg/m^2^)	43.2 ± 3.3	41.5 ± 2.7	44.2 ± 3.3	0.008
Waist circumference (cm)	126.0 ± 12.2	120.4 ± 10.8	129.1 ± 12.0	0.017
Fat mass (kg)	56.3 ± 7.1	54.9 ± 3.4	57.0 ± 8.4	0.227
Systolic blood pressure (mmHg)	132.0 ± 15.7	127.5 ± 13.9	134.4 ± 16.2	0.143
Diastolic blood pressure (mmHg)	88.1 ± 10.1	88.3 ± 9.0	88.1 ± 10.7	0.952
Heart rate (bpm)	71.9 ± 12.1	72.9 ± 12.0	71.4 ± 12.3	0.692
Triglycerides (mg/dL)	173.8 ± 68.8	172.7 ± 54.0	174.3 ± 76.6	0.939
HDL-cholesterol (mg/dL)	38.0 ± 6.9	39.3 ± 8.3	37.4 ± 6.0	0.354
LDL-cholesterol (mg/dL)	97.2 ± 35.8	110.2 ± 51.4	90.1 ± 21.2	0.139
Total cholesterol (mg/dL)	170.0 ± 42.3	184.1 ± 54.7	162.3 ± 32.2	0.147
Visceral adiposity index	3.9 ± 2.0	3.8 ± 1.5	3.9 ± 2.3	0.797
Glucose (mg/dL)	84.5 ± 19.0	78.5 ± 14.5	87.8 ± 20.6	0.468
Insulin (μU/mL)	9.8 ± 5.5	8.2 ± 3.9	10.8 ± 6.0	0.123
HOMA-IR	2.2 ± 1.7	1.7 ± 1.1	2.5 ± 1.9	0.101
Platelet count (×10^9^/L)	275.7 ± 67.3	330.2 ± 57.7	245.8 ± 52.2	<0.001
Albumin (g/L)	40.0 ± 2.8	41.2 ± 2.5	39.3 ± 2.7	0.026
Ferritin (ng/mL)	186.3 ± 173.3	135.9 ± 142.0	214.0 ± 184.6	0.137
ALAT (U/L)	43.4 ± 26.0	38.0 ± 25.7	46.4 ± 26.2	0.289
ASAT (U/L)	32.1 ± 16.2	28.1 ± 14.9	34.2 ± 16.6	0.214
ASAT/ALAT ratio	0.81 ± 0.27	0.87 ± 0.38	0.78 ± 0.18	0.262
GGT (U/L)	61.3 ± 45.8	51.9 ± 39.9	66.4 ± 48.6	0.299
Fatty liver index	97.9 ± 2.8	96.5 ± 3.7	98.6 ± 1.7	0.035
Creatinine (mg/dL)	0.78 ± 0.14	0.76 ± 0.12	0.79 ± 0.15	0.483
Glomerular filtration rate (mL/min/1.73 m^2^) ^3^	99.9 ± 14.3	104.0 ± 15.3	97.7 ± 13.4	0.149
hs-CRP (mg/L)	52.6 ± 39.1	49.6 ± 27.9	54.2 ± 44.4	0.698
Interleukin-6 (pg/mL)	10.7 ± 7.6	8.9 ± 6.5	11.7 ± 8.1	0.236

Abbreviations: ALAT, alanine aminotransferase; ASAT, aspartate aminotransferase; BMI, body mass index; GGT, γ-glutamyltransferase; and hs-CRP, high-sensitivity C-reactive protein. ^1^ Values are mean ± SD. * −1.455 < NFS < 0.675: *n* = 29; NFS > 0.675: *n* = 2. ^2^ *p*-values for inter-group comparisons (independent samples *t*-test). ^3^ Calculated using the Chronic Kidney Disease Epidemiology Collaboration (CKD-EPI) formula.

**Table 2:** Baseline anthropometric and clinical characteristics of the patients grouped using a HOMA-IR cut-off value of 2.0 ^1^.

	HOMA-IR < 2.0 (*n* = 28)	HOMA-IR ≥ 2.0 (*n* = 20)	*p*-Value ^2^
Age (years)	37.6 ± 9.3	43.6 ± 9.0	0.033
Height (m)	1.68 ± 0.1	1.70 ± 0.1	0.483
Body mass (kg)	119.8 ± 13.3	129.7 ± 18.1	0.035
BMI (kg/m^2^)	42.2 ± 3.0	44.6 ± 3.4	0.012
Waist circumference (cm)	121.2 ± 10.7	132.7 ± 11.2	0.001
Fat mass (kg)	56.0 ± 5.0	56.6 ± 9.1	0.810
Systolic blood pressure (mmHg)	127.1 ± 12.3	138.8 ± 17.5	0.009
Diastolic blood pressure (mmHg)	85.3 ± 8.8	92.1 ± 10.6	0.019
Heart rate (bpm)	69.3 ± 11.8	75.6 ± 11.7	0.076
Triglycerides (mg/dL)	151.9 ± 43.5	204.4 ± 85.6	0.008
HDL-cholesterol (mg/dL)	39.7 ± 7.3	35.8 ± 5.5	0.049
LDL-cholesterol (mg/dL)	92.8 ± 24.2	103.4 ± 47.6	0.318
Total cholesterol (mg/dL)	162.9 ± 28.2	180.0 ± 55.9	0.169
Visceral adiposity index	3.3 ± 1.2	4.7 ± 2.6	0.025
Glucose (mg/dL)	74.3 ± 8.9	98.9 ± 20.3	<0.001
Insulin (µU/mL)	6.2 ± 2.2	15.0 ± 4.4	<0.001
HbA1c	5.3 ± 0.4	6.2 ± 1.0	0.001
HOMA-IR	1.1 ± 0.4	3.7 ± 1.6	<0.001
ALAT (U/L)	31.8 ± 17.2	59.8 ± 27.9	<0.001
ASAT (U/L)	26.1 ± 11.0	40.4 ± 18.7	0.005
ASAT/ALAT ratio	0.89 ± 0.30	0.71 ± 0.19	0.022
GGT (U/L)	50.8 ± 38.9	75.9 ± 51.6	0.061
Platelet count (×10^9^/L)	281.8 ± 67.1	267.3 ± 68.5	0.468
Albumin (g/L)	39.8 ± 2.8	40.2 ± 2.8	0.570
Fatty liver index	96.9 ± 3.3	99.3 ± 0.5	0.001
NAFLD fibrosis score	−1.60 ± 1.1	−0.73 ± 1.2	0.013
Ferritin (ng/mL)	140.4 ± 108.8	250.6 ± 223.7	0.052
Creatinine (mg/dL)	0.77 ± 0.10	0.80 ± 0.18	0.623
Glomerular filtration rate (mL/min/1.73 m^2^) ^3^	100.6 ± 14.7	99.0 ± 14.0	0.697
hs-CRP (mg/L)	55.7 ± 30.3	48.3 ± 49.4	0.522
Interleukin-6 (pg/mL)	10.7 ± 7.4	10.7 ± 8.1	0.570

Abbreviations: ALAT, alanine aminotransferase; ASAT, aspartate aminotransferase; BMI, body mass index; GGT, γ-glutamyltransferase; and hs-CRP, high-sensitivity C-reactive protein. ^1^ Values are mean ± SD. ^2^ *p*-values for inter-group comparisons (independent samples *t*-test). ^3^ Calculated using the Chronic Kidney Disease Epidemiology Collaboration (CKD-EPI) formula.

**Table 3:** Baseline anthropometric and clinical characteristics of patients grouped according to ALAT activity (normal vs. abnormal; women ≤ 35 U/L vs. >35 U/L, men ≤ 50 U/L vs. >50 U/L) ^1^.

	Normal ALAT (*n* = 27)	Abnormal ALAT ^2^ (*n* = 21)	*p*-Value ^3^
Age (years)	39.4 ± 8.8	41.0 ± 10.6	0.573
Height (m)	1.68 ± 0.07	1.71 ± 0.10	0.326
Body mass (kg)	121.1 ± 12.6	127.5 ± 19.4	0.197
BMI (kg/m^2^)	42.9 ± 3.4	43.6 ± 3.3	0.469
Waist circumference (cm)	121.6 ± 11.3	131.6 ± 11.2	0.004
Fat mass (kg)	56.7 ± 5.6	55.6 ± 8.7	0.598
Systolic blood pressure (mmHg)	126.6 ± 9.6	138.9 ± 19.1	0.011
Diastolic blood pressure (mmHg)	85.3 ± 9.4	91.8 ± 9.8	0.023
Heart rate (bpm)	69.9 ± 12.5	74.5 ± 11.3	0.192
Triglycerides (mg/dL)	152.8 ± 55.6	200.7 ± 75.8	0.015
HDL-cholesterol (mg/dL)	39.1 ± 6.3	36.7 ± 7.4	0.224
LDL-cholesterol (mg/dL)	91.3 ± 18.2	104.9 ± 49.7	0.243
Total cholesterol (mg/dL)	160.9 ± 26.2	181.7 ± 55.3	0.124
Visceral adiposity index	3.5 ± 2.2	4.4 ± 1.4	0.100
Glucose (mg/dL)	79.3 ± 15.1	91.2 ± 21.7	0.029
Insulin (µU/mL)	8.6 ± 5.7	11.5 ± 4.8	0.064
HbA1c	5.5 ± 0.7	6.0 ± 1.0	0.081
HOMA-IR	1.8 ± 1.6	2.7 ± 1.6	0.056
ALAT (U/L)	25.4 ± 8.0	66.6 ± 22.5	<0.001
ASAT (U/L)	21.9 ± 7.9	45.1 ± 14.7	<0.001
ASAT/ALAT ratio	0.90 ± 0.30	0.70 ± 0.17	0.010
GGT (U/L)	46.9 ± 32.0	79.8 ± 54.4	0.020
Platelet count (×10^9^/L)	274.2 ± 51.6	277.6 ± 84.8	0.865
Albumin (g/L)	39.1 ± 2.4	41.1 ± 2.8	0.014
Fatty liver index	96.9 ± 3.4	99.2 ± 0.6	0.002
NAFLD fibrosis score	−1.23 ± 0.90	−1.25 ± 1.56	0.953
Ferritin (ng/mL)	123.3 ± 97.1	267.4 ± 214.6	0.008
Creatinine (mg/dL)	0.78 ± 0.10	0.78 ± 0.18	0.978
Glomerular filtration rate (mL/min/1.73 m^2^) ^4^	97.4 ± 15.0	103.2 ± 12.8	0.161
hs-CRP (mg/L)	56.1 ± 45.6	48.1 ± 29.1	0.489
Interleukin-6 (pg/mL)	11.3 ± 7.9	9.9 ± 7.5	0.540

Abbreviations: ALAT, alanine aminotransferase; ASAT, aspartate aminotransferase; BMI, body mass index; GGT, γ-glutamyltransferase; and hs-CRP, high-sensitivity C-reactive protein. ^1^ Values are mean ± SD. ^2^ Abnormal ALAT refers to >35 U/L for women and >50 U/L for men. ^3^ *p*-values for inter-group comparisons (independent samples *t*-test). ^4^ Calculated using the Chronic Kidney Disease Epidemiology Collaboration (CKD-EPI) formula.

**Table 4:** Anthropometric measures, blood pressure, and parameters related to the liver and glucose and lipid metabolism in patients with obesity after OAGB surgery, categorized according to their risk of advanced fibrosis ^1^.

	NFS ≤ −1.455 (*n* = 17)			NFS > −1.455 (*n* = 31)			
	Week 0	Week 12	Change	Week 0	Week 12	Change	*p*-Value ^2^
Body mass (kg)	116.5 ± 11.2	99.9 ± 10.6	−16.7 ± 3.8 ***	128.0 ± 17.1	108.4 ± 15.3	−19.6 ± 3.9 ***	0.017
BMI (kg/m^2^)	41.5 ± 2.7	35.6 ± 3.2	−5.9 ± 1.1 ***	44.2 ± 3.3	37.4 ± 3.1	−6.8 ± 1.3 ***	0.023
Fat mass (kg)	54.9 ± 3.4	41.5 ± 4.8	−13.4 ± 2.9 ***	57.0 ± 8.4	43.6 ± 8.4	−13.4 ± 3.9 ***	0.966
Waist circumference (cm)	120.4 ± 10.8	105.0 ± 10.7	−15.4 ± 3.8 ***	129.1 ± 12.0	113.4 ± 11.5	−15.7 ± 4.1 ***	0.808
Systolic BP (mmHg)	127.5 ± 13.9	119.8 ± 13.1	−7.7 ± 10.8 *	134.4 ± 16.2	119.9 ± 11.7	−14.6 ± 11.0 ***	0.042
Diastolic BP (mmHg)	88.3 ± 9.0	79.2 ± 8.6	−9.1 ± 8.8 ***	88.1 ± 10.7	79.7 ± 8.1	−8.4 ± 9.2 ***	0.801
Heart rate (mmHg)	72.9 ± 12.0	60.3 ± 8.1	−12.5 ± 10.9 ***	71.4 ± 12.2	61.9 ± 9.8	−9.5 ± 8.9 ***	0.308
Glucose (mg/dL)	78.5 ± 14.5	85.7 ± 9.8	7.1 ± 9.8 **	87.8 ± 20.6	88.0 ± 11.3	0.3 ± 15.6	0.108
Insulin (µU/mL)	8.2 ± 3.9	10.1 ± 5.0	1.9 ± 4.4	10.8 ± 6.0	9.0 ± 4.7	−1.7 ± 6.0	0.037
HbA1c	5.5 ± 0.5	5.3 ± 0.3	−0.2 ± 0.3 **	5.8 ± 1.0	5.2 ± 0.4	−0.6 ± 0.7 ***	0.012
HOMA-IR	1.7 ± 1.1	2.1 ± 1.1	0.5 ± 0.9	2.5 ± 1.9	2.0 ± 1.3	−0.5 ± 1.8	0.050
ALAT (U/L)	38.0 ± 25.7	29.4 ± 14.9	−8.6 ± 19.6	46.4 ± 26.2	39.2 ± 25.9	−7.2 ± 25.1	0.837
ASAT (U/L)	28.1 ± 14.9	20.8 ± 7.5	−7.3 ± 12.3	34.2 ± 16.6	28.8 ± 16.3	−5.4 ± 19.5	0.717
GGT (U/L)	51.9 ± 39.9	17.1 ± 7.8	−34.8 ± 36.2 **	66.4 ± 48.6	33.0 ± 50.2	−33.4 ± 41.5 ***	0.901
Fatty liver index	96.5 ± 3.7	71.5 ± 20.9	−25.0 ± 17.7 ***	98.6 ± 1.7	83.2 ± 15.5	−15.4 ± 14.2 ***	0.047
Triglycerides (mg/dL)	172.7 ± 54.0	115.5 ± 32.6	−57.2 ± 37.0 ***	174.3 ± 76.6	121.7 ± 63.1	−52.6 ± 42.6 ***	0.712
Total cholesterol (mg/dL)	184.1 ± 54.7	162.3 ± 35.5	−21.7 ± 28.2 **	162.3 ± 32.2	156.3 ± 31.5	−6.0 ± 21.4	0.035
HDL-cholesterol (mg/dL)	39.3 ± 8.3	43.2 ± 9.3	3.9 ± 4.5 **	37.4 ± 6.0	41.7 ± 5.0	4.4 ± 5.0 ***	0.732
LDL-cholesterol (mg/dL)	110.2 ± 51.4	96.1 ± 33.1	−14.2 ± 25.3 *	90.1 ± 21.2	90.2 ± 22.6	0.1 ± 16.1	0.021
Visceral adiposity index	3.8 ± 1.5	2.2 ± 0.9	−1.5 ± 0.9 ***	3.9 ± 2.3	2.5 ± 1.7	−1.5 ± 1.2 ***	0.852
hs-CRP (mg/L)	49.6 ± 27.9	6.1 ± 8.0	−43.5 ± 27.4	54.2 ± 44.4 ***	6.2 ± 5.8	−48.1 ± 43.6 ***	0.697
Ferritin (ng/mL)	135.9 ± 142.0	102.4 ± 83.2	−33.5 ± 71.6	214.0 ± 184.6	153.4 ± 138.4	−60.6 ± 109.7 **	0.365
Creatinine (mg/dL)	0.76 ± 0.11	0.72 ± 0.11	−0.04 ± 0.06 *	0.79 ± 0.15	0.75 ± 0.14	−0.04 ± 0.07 **	0.949
Glomerular filtration rate (mL/min/1.73 m^2^) ^3^	104.0 ± 15.3	110.0 ± 2.0	6.0 ± 10.3 *	97.7 ± 13.4	102.6 ± 12.8	4.9 ± 8.2 **	0.687

Abbreviations: ALAT, alanine aminotransferase; ASAT, aspartate aminotransferase; BMI, body mass index; BP, blood pressure; GGT, γ-glutamyltransferase; and hs-CRP, high-sensitivity C-reactive protein. * *p* < 0.05, ** *p* < 0.01, and *** *p* < 0.005 for intra-group comparisons (dependent samples *t*-test). ^1^ Values are mean ± SD. ^2.^*p*-values for inter-group comparisons of changes derived from the independent samples *t*-test. ^3^ Calculated using the Chronic Kidney Disease Epidemiology Collaboration (CKD-EPI) formula.

**Table 5:** Anthropometric measures, blood pressure, and parameters related to the liver and glucose and lipid metabolism in patients with obesity after OAGB surgery, categorized according to HOMA-IR ^1^.

	HOMA-IR < 2.0 (*n* = 28)			HOMA-IR ≥ 2.0 (*n* = 20)			
	Week 0	Week 12	Change	Week 0	Week 12	Change	*p*-Value ^2^
Body mass (kg)	119.8 ± 13.3	101.1 ± 11.5	−18.7 ± 3.9 ***	129.7 ± 18.1	111.4 ± 15.8	−18.3 ± 4.4 ***	0.745
BMI (kg/m^2^)	42.2 ± 2.9	35.7 ± 2.7	−6.6 ± 1.3 ***	44.6 ± 3.4	38.4 ± 3.2	−6.3 ± 1.3 ***	0.417
Body fat mass (kg)	56.0 ± 5.3	42.6 ± 6.0	−13.5 ± 2.9 ***	56.6 ± 9.1	43.3 ± 8.9	−13.3 ± 4.4 ***	0.898
Waist circumference (cm)	121.2 ± 10.7	104.8 ± 9.7	−16.5 ± 3.9 ***	132.7 ± 11.2	118.3 ± 9.8	−14.4 ± 3.8 ***	0.077
Systolic BP (mmHg)	127.1 ± 12.3	117.6 ± 11.7	−9.5 ± 10.7 ***	138.8 ± 17.5	123.0 ± 12.2	−15.8 ± 11.3 ***	0.056
Diastolic BP (mmHg)	85.3 ± 8.8	77.8 ± 8.1	−7.5 ± 8.5 ***	92.1 ± 10.6	81.9 ± 7.9	−10.3 ± 9.5 ***	0.299
Heart rate (bpm)	69.3 ± 11.8	60.4 ± 8.9	−8.9 ± 7.7 ***	75.6 ±11.7	62.7 ± 9.7	−12.9 ± 11.7 ***	0.160
Glucose (mg/dL)	74.3 ± 8.9	83.1 ± 7.7	8.9 ± 7.3 ***	98.9 ± 20.3	92.9 ± 11.9	−6.0 ± 16.8	<0.001
Insulin (µU/mL)	6.2 ± 2.2	7.5 ± 3.7	1.3 ± 3.6	15.0 ± 4.4	12.1 ± 4.9	−2.9 ± 7.7	0.010
HbA1c	5.3 ± 0.4	5.1 ± 0.3	−0.2 ± 0.2 **	6.2 ± 1.0	5.4 ± 0.4	−0.8 ± 0.7 ***	0.001
HOMA-IR	1.1 ± 0.4	1.5 ± 0.8	0.4 ± 0.8 *	3.7 ± 1.6	2.8 ± 1.3	−0.9 ± 2.1	0.003
ALAT (U/L)	31.8 ± 17.2	27.7 ± 15.9	−4.1 ± 17.2	59.8 ± 27.9	47.0 ± 26.7	−12.8 ± 29.2	0.200
ASAT (U/L)	26.1 ± 11.0	24.0 ± 15.6	−2.1 ± 15.4	40.4 ± 18.7	28.8 ± 12.1	−11.6 ± 18.4	0.058
GGT (U/L)	50.8 ± 38.9	17.3 ± 10.2	−33.6 ± 34.8 ***	75.9 ± 41.5	41.5 ± 60.5	−34.4 ± 45.9 **	0.947
NAFLD fibrosis score	−1.6 ± 1.1	−2.3 ± 1.3	−0.7 ± 0.7	−0.7 ± 1.2	−1.5 ± 1.1	−0.8 ± 0.7	0.633
Fatty liver index	96.9 ± 3.3	70.8 ± 18.4	−26.1 ± 15.7 ***	99.3 ± 0.5	90.6 ± 10.2	−8.7 ± 9.8 ***	<0.001
Triglycerides (mg/dL)	151.9 ± 43.5	102.1 ± 34.2	−49.8 ± 37.7	204.4 ± 85.6	143.9 ± 66.9	−60.5 ± 44.1	0.373
Total cholesterol (mg/dL)	162.9 ± 28.2	152.8 ± 26.8	−10.1 ± 22.1	180.0 ± 55.9	166.3 ± 39.0	−13.7 ± 28.9	0.620
HDL-cholesterol (mg/dL)	39.7 ± 7.3	44.2 ± 8.7	4.5 ± 5.3	35.8 ± 5.5	39.6 ± 5.4	3.8 ± 4.0	0.624
LDL-cholesterol (mg/dL)	92.8 ± 24.2	88.2 ± 21.1	−4.6 ± 16.8	103.4 ± 47.6	98.0 ± 32.5	−5.5 ± 25.7	0.891
Visceral adiposity index	3.3 ± 1.2	1.9 ± 0.8	−1.3 ± 0.8	4.7 ± 2.6	3.0 ± 1.9	−1.7 ± 1.4	0.301
hs-CRP (mg/L)	55.7 ± 30.3	6.2 ± 7.6	−49.5 ± 30.7 ***	48.3 ± 49.4	6.1 ± 5.0	−42.2 ± 47.6 ***	0.522
Ferritin (ng/mL)	140.4 ± 108.8	115.1 ± 88.6	−25.4 ± 70.9	250.6 ± 223.7	163.7 ± 158.0	−87.0 ± 119.4 **	0.048
Creatinine (mg/dL)	0.77 ± 0.10	0.71 ± 0.10	−0.06 ± 0.07 ***	0.79 ± 0.18	0.77 ± 0.16	−0.02 ± 0.08	0.089
Glomerular filtration rate (mL/min/1.73 m^2^) ^3^	100.6 ± 14.7	107.8 ± 11.5	7.2 ± 9.1 ***	99.0 ± 14.0	101.7 ± 14.2	2.7 ± 8.0	0.089

Abbreviations: ALAT, alanine aminotransferase; ASAT, aspartate aminotransferase; BMI, body mass index; BP, blood pressure; GGT, γ-glutamyltransferase; and hs-CRP, high-sensitivity C-reactive protein. * *p* < 0.05, ** *p* < 0.01, and *** *p* < 0.005 for intra-group comparisons (dependent samples *t*-test). ^1^ Values are mean ± SD. ^2^ *p*-values for inter-group comparisons (independent samples *t*-test). ^3.^ Calculated using the Chronic Kidney Disease Epidemiology Collaboration (CKD-EPI) formula.

## Data Availability

The data presented in this study are available on request from the corresponding author.

## Supplementary Materials

The following supporting information can be downloaded at: https://www.mdpi.com/article/10.3390/nu16183210/s1, Table S1: Anthropometric measures, blood pressure, and parameters related to the liver and glucose and lipid metabolism in patients with obesity after OAGB surgery supplemented with Pro + SM or Con + BM, categorized according to their risk of advanced fibrosis; Table S2: Anthropometric measures, blood pressure, and parameters related to the liver and glucose and lipid metabolism in patients with obesity after OAGB surgery supplemented with Pro + SM or Con + BM, categorized according to HOMA-IR.
