# Supplementary material for: Prognostic Characteristics of Metabolic Dysfunction-Associated Steatotic Liver in Patients with Obesity Who Undergo One Anastomosis Gastric Bypass Surgery: A Secondary Analysis of Randomized Controlled Trial Data
Source: Nutrients. 2024 Sep 23;16(18):3210. doi: 10.3390/nu16183210 (PMC11435136; doi:10.3390/nu16183210)
Supplement: Supplementary file 1 [file nutrients-16-03210-s001.zip › nutrients-3218255-supplementary.pdf]

Table S1. Anthropometric measures, blood pressure, and parameters related to the liver and glucose and lipid metabolism in patients with obesity after OAGB surgery supplemented with Pro+SM or Con+BM, categorized according to their risk of advanced fibrosis[1].

	**Specifically tailored treatment: Pro+SM**							**Basic treatment: Con+BM**						
	**NFS ≤ −1.455 (n = 6)**			**NFS > −1.455 (n = 19)**				**NFS ≤ −1.455 (n = 11)**			**NFS >− 1.455 (n = 12)**			
	**Week 0**	**Week 12**	**Change**	**Week 0**	**Week 12**	**Change**	***p*-Value[2]**	**Week 0**	**Week 12**	**Change**	**Week 0**	**Week 12**	**Change**	***p*-Value[2]**
Body mass (kg)	111.1 ± 6.0	96.0 ± 7.7	−15.1 ± 2.5***	129.5 ± 15.7	109.2 ± 14.8	−20.4 ± 3.3***	0.002	119.5 ± 12.4	102.0 ± 11.6	−17.5 ± 4.2***	125.4 ± 19.5	107.1 ± 16.6	−18.3 ± 4.6***	0.679
BMI (kg/m^2)	42.1 ± 2.6	36.4 ± 3.2	−5.7 ± 0.9***	44.4 ± 3.1	37.4 ± 3.2	−7.0 ± 1.1***	0.018	41.2 ± 2.8	35.2 ± 3.2	−6.0 ± 1.2***	43.9 ± 3.7	37.5 ± 3.1	−6.4 ± 1.4***	0.506
Waist circumference (cm)	116.3 ± 8.9	102.5 ± 10.8	−13.8 ± 3.4***	129.3 ± 13.1	113.8 ± 12.8	−15.4 ± 4.1***	0.399	122.6 ± 11.4	106.4 ± 10.8	−16.3 ± 3.9	128.8 ± 10.7	112.6 ± 9.5	−16.2 ± 4.4	0.952
Systolic BP (mmHg)	120.6 ± 13.6	116.8 ± 7.1	−3.7 ±13.1	135.2 ± 17.1	122.1 ± 10.9	−13.1 ± 11.7***	0.108	131.2 ± 13.1	121.4 ± 15.5	−9.9 ± 9.27**	133.2 ±15.5	116.3 ±12.5	−16.9 ± 9.8***	0.094
Diastolic BP (mmHg)	82.7 ± 9.3	77.7 ± 3.6	−4.9 ± 9.4	86.4 ± 11.3	80.0 ± 8.8	−6.4 ± 9.5**	0.751	91.3 ± 7.7	79.9 ± 10.5	−11.4 ± 8.0***	90.7 ± 9.6	79.1 ± 7.2	−11.7 ± 8.0***	0.932
Glucose (mg/dL)	74.8 ± 8.1	84.2 ± 6.6	9.3 ± 7.4*	90.3 ± 22.4	90.2 ± 12.7	−0.1 ± 17.4	0.215	80.6 ± 17.0	86.5 ± 11.4	5.9 ± 11.0	83.8 ±17.6	84.7 ± 7.9	0.8 ± 12.9	0.323
Insulin (µU/mL)	7.7 ± 2.3	11.4 ± 4.8	3.6 ± 5.5	10.7 ± 5.5	9.3 ± 4.4	−1.4 ± 6.1	0.081	8.5 ± 4.7	9.4 ± 5.1	0.9 ± 3.5	10.8 ± 7.0	8.6 ± 5.3	−2.13 ± 6.2	0.169
HbA1c	5.4 ± 0.4	5.2 ± 0.2	−0.2 ± 0.3	5.9 ± 1.0	5.3 ± 0.4	−0.6 ± 0.8**	0.159	5.6 ± 0.5	5.3 ± 0.4	−0.2 ± 0.3**	5.6 ± 0.8	5.2 ± 0.4	−0.5 ±0.5*	0.184
HOMA-IR	1.4 ± 0.5	2.4 ± 1.0	0.9 ± 1.2	2.6 ± 1.8	2.1 ± 1.3	−0.4 ± 2.0	0.124	1.8 ± 1.3	2.0 ± 1.2	0.2 ± 0.7	2.4 ± 2.0	1.9 ± 1.3	−0.6 ± 1.6	0.154
ALAT (U/L)	33.8 ± 19.8	26.8 ± 11.3	−7.0 ± 11.0	51.2 ± 29.2	41.4 ± 28.9	−9.8 ± 25.5	0.796	40.3 ± 29.1	30.7 ± 16.9	−9.5 ± 23.5	38.8 ± 19.3	35.8 ± 21.0	−3.0 ± 24.8	0.525
ASAT (U/L)	24.3 ± 7.4	17.3 ± 3.0	−7.0 ± 5.7*	37.0 ± 17.1	27.4 ± 12.6	−9.5 ± 12.3**	0.635	30.2 ± 17.7	22.7 ± 8.6	−7.5 ± 15.0	29.9 ± 15.6	31.1 ± 21.3	1.2 ± 26.7	0.357
GGT (U/L)	77.3 ± 50.4	17.3 ± 7.1	−60.0 ± 43.4*	68.7 ± 51.4	37.8 ± 62.9	−30.9 ± 46.5*	0.188	38.0 ± 26.0	16.9 ± 8.5	−21.0 ± 23.9*	62.8 ± 45.7	25.4 ± 16.9	−37.3 ± 33.8**	0.201
Fatty liver index	97.1 ± 4.2	75.3 ± 19.6	−21.9 ± 15.8*	98.7 ± 1.4	81.8 ± 16.6	−16.9 ± 15.5***	0.505	96.1 ± 3.6	69.4 ± 22.1	−26.7 ± 19.1***	98.5 ± 2.2	85.4 ± 14.1	−13.1 ± 12.1**	0.053
NFS	−2.89 ± 1.51	−3.69 ± 1.59	−0.80 ± 0.82	−0.63 ± 0.80	−1.59 ± 0.95	−0.96 ± 0.56***	0.585	−2.14 ± 0.53	−2.64 ± 0.86	−0.50 ± 0.89	−0.54 ± 0.73	−1.10 ± 0.70	−0.56 ± 0.53**	0.844

Triglycerides (mg/dL)	182.8 ± 63.7	125.3 ± 21.0	−57.5 ± 52.5*	192.0 ± 88.5	124.4 ± 70.3	67.6 ± 38.2***	0.661	167.2 ± 50.4	110.2 ± 37.3	−57.0 ± 28.4***	146.3 ± 42.3	117.4 ± 52.4	−28.9 ± 39.6***	0.066
Total-cholesterol (mg/dL)	206.9 ± 81.8	172.2 ± 48.4	−34.7 ± 36.4	168.6 ± 34.3	156.4 ± 35.0	−12.3 ± 22.6*	0.081	171.6 ± 30.9	157.0 ± 27.4	−14.7 ± 21.4*	152.4 ± 26.9	156.2 ± 26.5	3.80 ± 15.4	0.026
HDL-cholesterol (mg/dL)	35.3 ± 5.6	39.7 ± 8.1	4.3 ± 3.1*	37.6 ± 6.8	42.6 ± 6.6	5.0 ± 4.6***	0.747	41.5 ± 8.9	45.1 ± 9.7	3.6 ± 5.2*	36.9 ± 4.7	40.3 ± 7.5	3.4 ± 5.6	0.924
LDL-cholesterol (mg/dL)	135.0 ± 77.1	107.5 ± 49.8	−27.5 ± 30.7	92.6 ± 21.3	88.8 ± 25.8	−3.7 ± 18.1	0.027	96.7 ± 26.2	89.8 ± 19.8	−6.9 ± 19.7	86.2 ± 21.3	92.3 ± 17.3	10.3 ± 2.96	0.056
VAI	4.5 ± 2.0	2.7 ± 1.1	−1.8 ± 1.3*	4.3 ± 2.8	2.5 ± 1.9	−1.8 ± 1.2***	0.948	3.4 ± 0.9	2.0 ± 0.6	0.7 ± 0.2***	3.4 ± 1.1	2.5 ± 1.4	1.0 ± 0.3*	0.185
hs-CRP (mg/L)	39.1 ± 15.0	5.0 ± 2.3	−34.1 ± 15.5**	44.1 ± 23.9	6.5 ± 6.6	−37.6 ± 24.3***	0.744	55.3 ± 32.1	6.7 ± 10.0	−48.6 ± 31.6***	70.2 ± 63.1	5.5 ± 4.4	−64.7 ± 61.0**	0.443
Ferritin (ng/mL)	124.5 ± 38.0	102.2 ± 27.6	−22.3 ± 26.8	218.7 ± 216.0	145.8 ± 133.9	−72.9 ± 110.4**	0.284	142.1 ± 177.3	102.5 ± 103.4	−39.6 ± 87.9	206.6 ± 128.3	165.4 ± 150.4	−41.2 ± 110.5	0.971
Creatinine (mg/dL)	0.72 ± 0.10	0.70 ± 0.08	−0.02 ± 0.03	0.80 ± 0.16	0.75 ± 0.16	−0.05 ± 0.08*	0.231	0.79 ± 0.12	0.73 ± 0.12	−0.06 ± 0.09	0.78 ± 0.14	0.74 ± 0.12	−0.04 ± 0.06*	0.603
Glomerular filtration rate (mL/min/1.73 m^2)[3]	109.0 ± 17.1	111.8 ± 16.6	2.7 ± 4.7	98.5 ± 11.7	103.5 ± 10.7	5.0 ± 9.6*	0.589	101.2 ± 14.4	109.1 ± 9.4	7.8 ± 12.1	96.5 ± 16.3	101.4 ± 16.1	4.9 ± 5.6*	0.456

Abbreviations: ALAT, alanine aminotransferase; ASAT, aspartate aminotransferase; BM, basic care micronutrient; BMI, body mass index; BP, blood pressure; Con, control; GGT, γ-glutamyltransferase; hs-CRP, high-sensitivity C-reactive protein; NFS, NAFLD fibrosis score; OAGB, one anastomosis gastric bypass; Pro, probiotic; SM, specific micronutrient; VAI, visceral adiposity index. * $P<0.05$, ** $P<0.01$, *** $P<0.005$ for intra-group comparisons (dependent samples *t*-test). [1] Values are mean ± SD. [2] *P*-values for inter-group comparisons of changes derived from the independent samples *t*-test. [3] Calculated using the Chronic Kidney Disease Epidemiology Collaboration (CKD-EPI) formula.

Table S2. Anthropometric measures, blood pressure, and parameters related to the liver and glucose and lipid metabolism in patients with obesity after OAGB surgery supplemented with Pro+SM or Con+BM, categorized according to HOMA-IR[1].

	**Specifically tailored treatment: Pro+SM**							**Basic treatment: Con+BM**						
	**HOMA-IR < 2.0 (n = 14)**			**HOMA-IR ≥ 2.0 (n = 11)**				**HOMA-IR < 2.0 (n = 14)**			**HOMA-IR ≥ 2.0 (n = 9)**			
	**Week 0**	**Week 12**	**Change**	**Week 0**	**Week 12**	**Change**	***p*-Value[2]**	**Week 0**	**Week 12**	**Change**	**Week 0**	**Week 12**	**Change**	***p*-Value[2]**
Body mass (kg)	119.9 ± 15.0	101.2 ± 13.0	−18.8 ± 4.1***	131.7 ± 15.4	112.2 ± 14.5	−19.5 ± 3.7***	0.667	119.7 ± 11.9	101.1 ± 10.4	−18.6 ± 3.9***	127.2 ± 21.7	110.3 ± 18.2	−16.9 ± 4.9***	0.356
BMI (kg/m²)	42.6 ± 2.3	35.9 ± 2.0	−6.7 ± 1.3***	45.4 ± 3.4	38.7 ± 3.7	−6.7 ± 1.1***	0.979	41.9 ± 3.5	35.4 ± 3.3	−6.5 ± 1.2***	43.8 ± 3.4	38.0 ± 2.8	−5.8 ± 1.4***	0.21
Waist circumference (cm)	120.1 ± 11.1	104.4 ± 10.5	−15.6 ± 3.7***	133.9 ± 12.0	119.6 ± 11.2	−14.3 ± 4.3***	0.397	122.4 ± 10.5	105.1 ± 9.3	−17.3 ± 4.2***	131.2 ± 10.7	116.7 ± 8.2	−14.6 ± 3.5***	0.118
Systolic BP (mmHg)	124.3 ± 12.4	117.2 ± 9.3	−7.1 ± 10.8*	141.2 ± 18.5	125.4 ± 9.9	−15.7 ± 13.3**	0.084	129.9 ± 12.0	117.9 ± 14.0	−12.0 ± 10.5***	135.9 ± 16.9	120.0 ± 14.5	−15.9 ± 9.1***	0.362
Diastolic BP (mmHg)	81.1 ± 8.6	76.1 ± 7.2	−5.0 ± 9.6	91.1 ±11.0	83.8 ± 6.7	−7.3 ± 9.1*	0.544	89.5 ± 6.9	79.5 ± 9.0	−10.0 ± 6.6***	93.4 ± 10.7	79.5 ± 9.0	−13.8 ± 9.3**	0.262
Glucose (mg/dL)	75.1 ± 6.0	84.6 ± 6.5	9.5 ± 7.5***	101.1 ± 24.2	93.9 ± 14.9	−7.2 ± 19.3	0.018	73.4 ± 11.2	81.6 ± 8.8	8.2 ± 7.3***	96.1 ± 15.4	91.7 ± 7.6	−4.4 ± 14.2	0.010
Insulin (µU/mL)	6.6 ± 2.1	8.9 ± 4.3	2.3 ± 4.3	14.3 ± 4.3	10.9 ± 4.6	−3.4 ± 7.0	0.019	5.8 ± 2.2	6.1 ± 2.3	0.3 ± 2.4	15.8 ± 4.7	13.5 ± 5.1	−2.2 ± 7.7	0.261
HbA1c	5.3 ± 0.3	5.2 ± 0.2	−0.2 ± 0.2*	6.4 ± 1.2	5.4 ± 0.5	−1.0 ± 0.8**	0.008	5.4 ± 0.5	5.1 ± 0.4	−0.2 ± 0.2***	6.0 ± 0.8	5.4 ± 0.4	−0.5 ± 0.5*	0.072
HOMA-IR	1.2 ± 0.4	1.9 ± 0.9	0.6 ± 1.0*	3.7 ± 1.7	2.6 ± 1.4	−1.1 ± 2.3	0.020	1.1 ± 0.5	1.2 ± 0.5	0.2 ± 0.5	3.8 ± 1.5	3.1 ± 1.5	−0.7 ± 1.8	0.189
ALAT (U/L)	33.2 ± 21.0	24.8 ± 8.6	−8.4 ± 15.5	64.6 ± 26.2	54.6 ± 31.9	−10.1 ± 30.4	0.860	30.4 ± 13.0	30.6 ± 20.8	0.3 ± 18.2	53.8 ± 30.3	37.7 ± 15.6	−16.1 ± 29.1	0.110
ASAT (U/L)	26.6 ± 11.7	20.1 ± 5.1	−6.4 ± 9.8*	43.3 ± 16.6	31.2 ± 15.1	−12.1 ± 12.2**	0.210	25.6 ± 10.7	27.9 ± 21.1	2.2 ± 18.9	36.9 ± 21.4	25.9 ± 6.6	−11.0 ± 24.8	0.162
GGT (U/L)	64.6 ± 42.2	16.9 ± 7.0	−47.7 ± 38.6***	78.6 ± 60.3	53.2 ± 80.4	−25.4 ± 54.5	0.242	37.0 ± 10.8	17.6 ± 12.9	−19.4 ± 24.3*	72.6 ± 41.8	27.2 ± 14.1	−45.3 ± 32.5**	0.040
Fatty liver index	97.6 ± 3.0	73.3 ± 16.9	−24.3 ± 14.7***	99.3 ± 0.6	89.1 ± 13.4	−10.2 ± 12.9*	0.019	96.1 ± 3.6	68.3 ± 20.0	−27.8 ± 17.0***	99.2 ± 0.5	92.4 ± 4.1	−6.8 ± 3.7***	0.001

NFS	−1.68 ± 1.50	−2.53 ± 1.51	−0.85 ± 0.57***	−0.53 ± 0.94	−1.54 ± 1.15	−1.01 ± 0.69***	0.518	−1.51 ± 0.67	−2.05 ± 1.14	−0.54 ± 0.74*	−0.98 ± 1.4	−1.50 ± 1.0	-0.5 ± 0.7	0.946
Triglycerides (mg/dL)	158.6 ± 43.5	104.9 ± 28.4	−53.7 ± 38.0***	229.6 ± 102.9	149.8 ± 82.6	−79.7 ± 42.0***	0.188	145.2 ± 44.1	99.4 ± 40.1	−45.9 ± 38.4***	173.6 ± 47.3	136.7 ± 44.6	−36.9 ± 35.8*	0.581
Total-cholesterol (mg/dL)	165.5 ± 30.6	150.5 ± 24.6	−15.0 ± 20.9*	193.5 ± 66.6	172.5 ± 49.1	−20.9 ± 35.0	0.604	160.3 ± 26.4	155.2 ± 29.6	−5.1 ± 22.8	163.6 ± 36.3	158.7 ± 21.7	−4.9 ± 17.2	0.985
HDL-cholesterol (mg/dL)	38.4 ± 6.1	43.4 ± 7.4	5.0 ± 4.5**	35.4 ± 6.9	40.0 ± 6.0	4.6 ± 4.2**	0.838	40.9 ± 8.4	44.9 ± 10.0	4.0 ± 6.2*	36.2 ± 3.6	39.0 ± 4.9	2.8 ± 3.8	0.603
LDL-cholesterol (mg/dL)	95.4 ± 26.7	86.1 ± 23.1	−9.3 ± 14.5*	112.2 ± 59.2	102.6 ± 41.6	−9.6 ± 32.3	0.971	90.3 ± 22.1	90.4 ± 19.5	0.1 ± 18.2	92.7 ± 27.6	92.3 ± 16.9	−0.3 ± 14.6	0.954
VAI	3.5 ± 1.5	2.0 ± 1.0	−1.5 ± 0.9***	5.5 ± 3.2	3.2 ± 2.3	−2.3 ± 1.4***	0.088	3.0 ± 0.7	1.8 ± 0.6	−1.2 ± 0.7***	3.9 ± 1.2	2.9 ± 1.5	−1.0 ± 1.1*	0.599
hs-CRP (mg/L)	49.5 ± 25.0	6.0 ± 6.2	−43.6 ± 25.7***	34.5 ± 14.1	6.4 ± 5.7	−28.1 ± 13.7***	0.084	61.8 ± 34.6	6.4 ± 9.1	−55.4 ± 35.0***	65.1 ± 70.4	5.6 ± 4.2	−59.5 ± 67.4*	0.851
Ferritin (ng/mL)	144.3 ± 114.8	114.3 ± 99.0	−29.0 ± 53.3	262.0 ± 251.1	160.8 ± 139.6	−101.2 ± 128.6*	0.105	136.6 ± 106.6	114.9 ± 80.6	−21.7 ± 87.1	236.7 ± 199.0	167.1 ± 186.8	−69.6 ± 112.0	0.263
Creatinine (mg/dL)	0.78 ± 0.11	0.72 ± 0.11	−0.07 ± 0.06***	0.78 ± 0.20	0.77 ± 0.18	−0.01 ± 0.08	0.076	0.77 ± 0.10	0.71 ± 0.10	−0.06 ± 0.08*	0.82 ± 0.17	0.78 ± 0.14	−0.04 ± 0.07	0.589
Glomerular filtration rate (mL/min/1.73 m^2)[3]	101.7 ± 14.7	108.9 ± 12.6	7.3 ± 7.6**	100.3 ± 12.5	101.1 ± 11.4	0.8 ± 8.7	0.061	99.6 ± 15.1	106.7 ± 10.7	7.0 ± 10.7*	97.4 ± 16.3	102.5 ± 17.7	5.1 ± 6.7	0.633

Abbreviations: ALAT, alanine aminotransferase; ASAT, aspartate aminotransferase; BM, basic care micronutrient; BMI, body mass index; BP, blood pressure; Con, control; GGT, γ-glutamyltransferase; hs-CRP, high-sensitivity C-reactive protein; NFS, NAFLD fibrosis score; OAGB, one anastomosis gastric bypass; Pro, probiotic; SM, specific micronutrient; VAI, visceral adiposity index. * $P<0.05$, ** $P<0.01$, *** $P<0.005$ for intra-group comparisons (dependent samples *t*-test). [1] Values are mean ± SD. [2] *P*-values for inter-group comparisons of changes derived from the independent samples *t*-test. [3] Calculated using the Chronic Kidney Disease Epidemiology Collaboration (CKD-EPI) formula.
